# Investigating the Vinblastine Induced-Chromosomal Abnormality in the Already Gamma Irradiated L929 Cell Line Using Micronucleus Assay in Cytokinesis Blocked Binucleated Cells

**DOI:** 10.31557/APJCP.2019.20.4.1045

**Published:** 2019

**Authors:** Zahra Mohammadi, Farhang Haddad, Maryam M Matin, Shokouhozaman Soleymanifard

**Affiliations:** 1 *Department of Biology, Faculty of Sciences,*; 2 *Department of Biology and Institute of Biotechnology, Cell and Molecular Biotechnology Research Group, Ferdowsi University of Mashhad,*; 3 *Medical Physics Research Center, Mashhad University of Medical Sciences, Mashhad, Iran. *

**Keywords:** Gamma irradiation, Vinblastine, Micronucleus assay, L929 cell

## Abstract

**Objectives:**

Vast number of studies show the relationship between aneuploidy and cancer. Ionizing radiation in addition to induce all kinds of damages to the cells and structure of chromosomes, is also able to induce aneuploidy through direct damages to chromosome division apparatus. Also irradiation of the cells induces mutations in several genes which might be involved in cell division fidelity and play a role in reversing the effect of aneugens. Therefore, irradiation of cells and tissues might produce sensitivity to agents with aneugenic capability in irradiated cells.

**Methods:**

To investigate the persistent genomic effect of ionizing irradiation on chromosomal instability, L929 cells were gamma irradiated with the dose of 2 Gy. Cells were left to recover from the harmful effect of irradiation. They were treated with low dose of vinblastine (0.5 ng.ml-1) 72h post-gamma irradiation. Finally, the induced chromosomal abnormalities were scored using micronucleus assay in cytokinesis-blocked binucleated cells (MnBi).

**Results:**

Irradiation-recovered L929 cells treated with vinblastine showed a statistically higher frequency of MnBi compared to non-irradiated and vinblastine treated cells.

**Conclusion:**

The results indicate that gamma irradiation, in addition to direct induction of chromosomal damages, is also able to create persisting genomic sensitivity in the cells to chromosomal instability, which is detectable when exposed to the second stimulus.

## Introduction

Aneuploidy is the result of chromosome malsegregation during cell division. Aneuploid cells gain or loss one or more chromosome(s). Aneuploidy is responsible for a wide range of problems in human life. It is the main cause for many abortions, newborn abnormalities, sterility (Hassold et al., 2007), and cancer (Cimini and Degrassi, 2005). The important role of aneuploidy in cancer has been suggested in many studies (Duesberg and Li, 2003; Gordon et al., 2012; Giam and Rancati, 2015). 

Aneuploidy, by unbalancing the expression of several genes, induces tumor formation through disturbing the cell equilibrium. Damages to the cells normal status, in turn, induce more abnormal chromosome segregation during cell divisions (Passerini and Storchova, 2016). The relationship between aneuploidy and cancer is so strong that it is suggested to be used as a biomarker for integrated chemical assessments of carcinogenicity (Mandrioli et al., 2016). The proposed relationship between aneuploidy and cancer explains why some cancers are caused by non-mutagenic carcinogens and are chromosomally unstable (Duesberg et al., 2006). Investigating the reasons for the induction of aneuploidy is the interest of many studies. The more understanding about aneuploidy, the more effective therapeutic procedures would be developed (Tanaka and Hirota, 2016). Environmental stimuli with a positive effect on aneuploidy induction are being studied in mutagenesis investigations (Jin et al., 2015).

Ionizing radiation has always been known as a strong clastogenic factor. Moreover, in some studies, its aneugenic effect has also been suggested. The results of different experiments show that in addition to clastogenic effect, it is also able to induce aneuploidy in irradiated cells (Ponsa, 2001; Tateno et al., 2011; Cho et al., 2015). Ionizing radiation directly induces chromosome malsegregation through imposition damages to spindle poles and centrosome integrity (Sgura, 2001; Maxwell et al., 2008). It might be also able to induce damages to the genes involved in chromosome segregation during cell cycles. These damages are created through its mutagenic capability, mediated by induced oxidative stress, which indirectly leads to aneuploidy (Ikawa-Yoshida et al., 2013).

The risk of the second tumor formation is considerably high in cancer treatment protocols, which include radiotherapy (Imaoka et al., 2016). The susceptibility of cancer patients, who have received radiation, to the increased risk of second cancer suggests the higher sensitivity of the irradiated normal cells to other stimuli capable to induce genomic instability.

To study the indirect effect of ionizing radiation in chromosome malsegregation, we investigate the fidelity of chromosome segregation in the cells recovered from gamma-irradiation using a known strong aneugen, vinblastine.

## Materials and Methods

*Cell line*

The L929 cell line, passage 13-15, was used in this experiment. The cells were cultured in LG DMEM (Gibco) completed with 10% FBS (Gibco) and left in 37^o^C and 5% CO_2_ until needed. Cells were sub-cultured every 72h in the ratio of 1 to 5. Cell culture was performed in duplicate for each treatment and its corresponding control.

*Irradiation*

Gamma irradiation of the cells with the rate of 0.99Gy.Min-1 was performed 24h post culture initiation at the final dose of 2Gy in T25 flask (60Co radiation therapy, Therateron, Canada). Irradiated cells, as well as controls, were harvested 24, 48, 72, and 96h post-irradiation. Culture medium replacement was gently performed at 72h post-culture initiation only for the culture harvested 96h post-irradiation.

Vinblastine treatment

Vinblastine sulfate (Gedeon Ichter Ltd.) was dissolved in distilled water to the final concentration of 1µg.ml^-1^. Cells were treated with two doses of 0.5 and 1.5ng.ml^-1 ^of vinblastine for 24h. When needed, the vinblastine treatment was performed 72h after irradiation with a dose of 0.5ng.ml-1 for further 24h. The culture medium was gently replaced with a medium containing cytochalasin-B after this period. During the entire experiment time, sub-culturing was performed every 72h.

*Cell harvest*

Cell harvest was performed according to suggested procedure (Fenech, 2000) with slight modifications. Cytochalasin-B with the final concentration of 4µg.ml-1 was added to the cultures 20h before harvest. Cells were centrifuged and washed twice with fixator of 9:1 methanol: acetic acid. Few cell drops were placed on the clean microscopic slides and left aside to air dry.

Slides were stained using 20% Giemsa for 20 min.

*Scoring*

Scoring took place at a 1,000x magnification. In this study, cells with two detached nuclei were scored as a binucleated cell (Bi). For each treatment and control, at least three slides were coded, and on each slide, at least 500 Bi were blindly scored. In all Bi scored, cells harboring small unattached nucleus were considered as a micronucleated binucleate cell (MnBi). The frequency of MnBi in each treatment and control was calculated as:

*Frequency of MnBi= Number of MnBi/ Total Number of Bi scored*

*MTT assay*

Irradiated cells, as well as untreated control cells, were cultured in 96 wells culture plates. Cell viability test was performed for control and irradiated cells harvested 24, 48, 72, and 96h post-irradiation. Next, 100µg of a tetrazolium salt (Sigma), dissolved in PBS, was added to 200µl of cell culture medium and left for 6h in 37^o^C. The medium of each well was discarded and 150µl of DMSO was added to each well. Light absorbance of each well at 545 nm wavelength was recorded using Eliza reader (AWASENESS). The light absorbance value of each well was compared to that of the control and its graph was prepared.

*Statistical analysis*

The statistical analysis was performed using software MINITAB version 14. The differences between treated and control groups and also between treated groups were analyzed by one-way analysis of variance (ANOVA).

## Results

**Figure 1:**
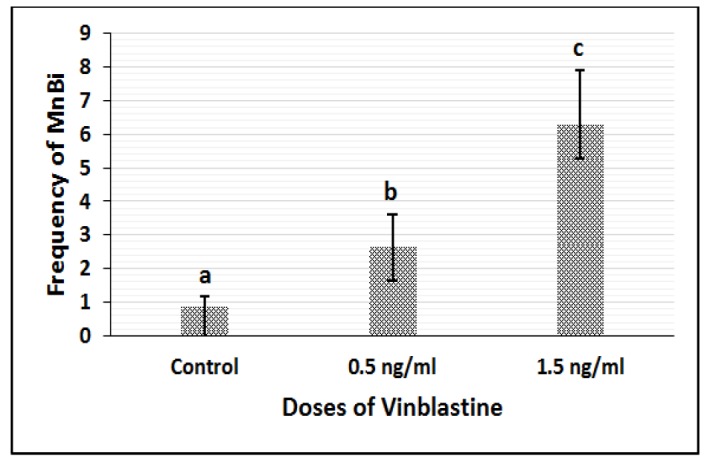
Frequency of MnBi in Cells Treated with Two Doses of Vinblastine. a, b, c statistical differences between groups (P<0.01)

Vinblastine treatment:Data for vinblastine treatment are presented in Figure 1. Treating the L929 cells with two different doses of vinblastine significantly increased the frequency of MnBi compared to control (P<0.01). The dose of 1.5ng.ml-1 of vinblastine showed a stronger effect in inducing Mn compared to 0.5 ng.ml-1 (P<0.01).

**Figure 2:**
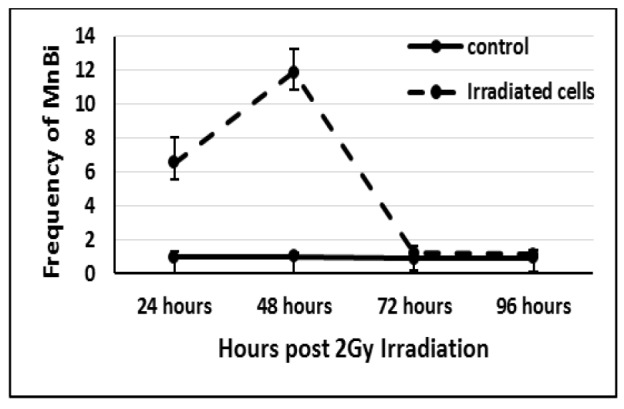
Recovery of the Cells after 2 Gy Gamma Irradiation

**Figure 3:**
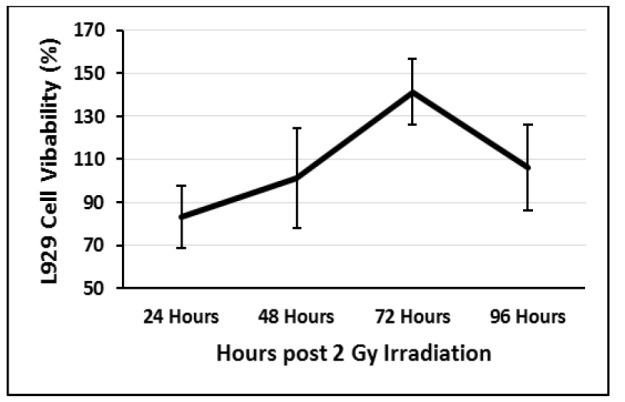
L929 Cells Viability at Different Time Interval after 2 Gy Gamma Irradiation

**Figure 4:**
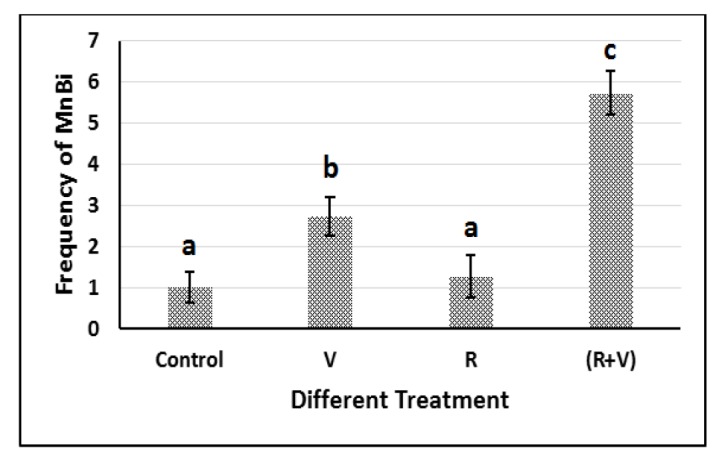
Frequency of MnBi in Different Treatments. R, Recovered cells 72 hours post-irradiation; V, Vinblastine treated cells. a, b, c statistical differences between groups (P<0.01)

*2 Gy Gamma irradiation and cell recovery*

The L929 cells were treated with 2Gy Gamma irradiation and harvested at different time points. Figure 2 shows the time-dependent reduction of Mn frequency in irradiated cells. The highest frequency of MnBi was at 48h post-treatment. At 72h after irradiation, the frequency of MnBi was back to the normal baseline and showed no significant differences with control.

The cell viability of gamma-irradiated cells was also analyzed (Figure 3). Irradiated cells showed a significant decrease in cell viability at 24h post-irradiation and a time-dependent cell recovery occurred. Cells were significantly recovered and backed to normal percent of viability at 72h post-irradiation.

*Vinblastine treatment of Irradiated cells*

Data of different treatment combination of the L929 cells are shown in Fig. 4. Cells treated with 0.5 ng.ml^-1 ^vinblastine showed a significantly higher frequency of MnBi compared to that of the control (P<0.01). However, Cells recovered after 72h from 2 Gy Gamma irradiation showed no increase in the frequency of MnBi compared to control. Treatment of 72h post-irradiation recovered cells with a 0.5 ng.ml-1 dose of vinblastine significantly induced the frequency of MnBi compared to control as well as other treatments. 

## Discussion

Aneuploidy is the result of chromosome malsegregation during cell division. Cancer cells exhibit some sort of aneuploidy and their severity is associated with the degree of cancer (Sen, 2000; Duesberg et al., 2006). Due to the proposed important role of aneuploidy in tumor formation and cancer (Devi and Satyamitra, 2005; Mandrioli et al., 2016), studying the causes behind aneuploidy formation and potential triggering factors of aneuploidy is of paramount importance. Studies show that ionizing radiation in addition to its clastogenic effect is able to induce aneuploidy (Maxwell et al., 2008; Jin et al., 2015). Its ability to cause chromosome loss or nondisjunction is believed to be the result of direct damages to chromosome segregation apparatus and deregulating the centrosome synthesis during cell division (Maxwell et al., 2008).

Irradiation-induced aneuploidy at several cell cycle post irradiation has not been investigated in other studies. In the present work, we investigated the effect of persisting genetic damages caused by ionizing radiation in chromosome instability during cell division. The clastogenic ability of ionizing radiation has been established in several studies. Irradiation can cause chromatid /chromosome breaks in treated cells as a result of its direct effect on DNA or by producing free radical capable of interacting with DNA and finally causing breaks in chromosomes (Suto et al., 2015; Pathak et al., 2016). In this study, 2 Gy gamma irradiation resulted in a significant increase in MnBi, with the highest frequency at 48h post-irradiation. The increase in the frequency of MnBi was the result of increased levels of breaks in chromosomes structure, which could be seen as micronucleus in binucleated cells. Gamma irradiation of human lymphocytes in vitro also increased the centromere-negative micronuclei, which indicate the clastogenic capability of gamma irradiation (Vral et al., 1997). One Gy Gamma irraidaiton of TK6 human lymphoblastoid cell line also led to the almost same frequency of micronuclei binucleated cells as our study (Yamamoto et al., 2017).

When micronuclei-inducing stimuli are removed, the induced micronuclei will be unstable and their frequency will be reduced because of either losing them during cell divisions or integration into main nuclei (Leach and Jackson-Cook, 2004). In this study, the frequency of Gamma-induced micronuclei backed to normal 72h post-irradiation. Twenty-four hours after that, no increase in the frequency of MnBi was detected, suggesting that cells were in some equilibrium after a harmful event. Also, cell viability analysis confirms the ability of the cells to recover from the harmful effect of gamma irradiation after 72h and maintain the same percent of viable cells as untreated cells. Time-response investigation of gamma-irradiated human lymphoblastoid also showed an increase and decrease in the frequency of micronucleus at first and the third cell cycle post-irradiation, respectively (Ramirez et al., 1999). The cell cycle time of the L929 cells is 36 hours (Baca et al., 1985). So, in our experiment the L929 cells showed and increase and decrease of micronucleus at first and second cell cycle respectively. The time response difference between our data and the result of that study could be related to the higher ability of L929 cells to adapt with any disturbances compared to human lymphoblastoid cell lines. Parasidic infection of L929 cells also did not change the cell cycle time (Baca et al., 1985).

In this study, the chromosome instability was induced by vinblastine (C46H58N4O9), which is a Vinca alkaloid with the confirmed aneugenic potential in several in vitro and in vivo studies (Leopardi et al., 2002; Cammerer et al., 2010). Its aneugenic ability even at low doses has been already confirmed (Huber et al., 1996; Marshall et al., 1996; Zijno et al., 1996). Treatment of the cells with vinblastine in this study also led to an increase in MnBi frequency. Due to the aneugenic capability of vinblastine, the increased level of Mn in treated cells with two doses of vinblastine was probably related to the whole chromosome loss during cell divisions. Micronucleus assay in binucleated cells would reveal the chromosome loss for the whole or a part of the chromosome as micronucleus in the cytoplasm. In this regard, the only way to distinguish the Mn caused by whole chromosome loss or part of the chromosome is to perform centromere labeling either by centromeric DNA probes or by anti-kinetochore antibodies. In many studies, treating the cells with different doses of vinblastine has been led to significant increase in centromere positive Mn which suggested induced chromosome loss and aneuploidy in those cells (Huber et al., 1996; Marshall et al., 1996; Zijno et al., 1996).

Treating the cells recovered from gamma irradiation with vinblastine led to the statistically significant increase in the frequency of MnBi compared to the vinblastine treated /non-irradiated cells. This increase shows the susceptibility of the irradiated cells to the aneugenic activity of vinblastine. Although the irradiated cells seem to be recovered from chromosomal effects of irradiation, judged by baseline frequency of MnBi, but they seem to be genetically damaged. Such damages result in a higher sensitivity of the cells to vinblastine treatment so that it is able to induce chromosome segregation irregularity in these cells more profoundly.

Several genes are involved in controlling the fidelity of chromosome segregation during cell division such as BUB3, AURKA, DYNC1I1, DCTN2, and CHFR. Any mutations or changes in these genes, which might influence their expression or function of their product, will lead to malsegregation of chromosomes such as loss and non-disjunction (Ertych et al., 2016; Krause et al., 2016). Mutation in BUB3, an important member of spindle assembly checkpoint, leads to mosaic aneuploidy in affected individuals and a higher risk of colorectal cancer (de Voer et al., 2013).

In addition to its clastogenic damages to genetic material, ionizing irradiation is able to induce gene mutations in irradiated cells (Adewoye et al., 2015; Shahid et al., 2015). As other genes in the cells, the ones involved in chromosome segregation and those involved in controlling cell division fidelity might be targeted by the ionizing irradiation. These mutated genes could not perform their duty during cell division. In these cells when there are no harmful stimuli which might lead to chromosome loss or non-disjunction, no disruption in chromosome migration is monitored. However, when treated with second agent capable to induce chromosomal malsegregation, mutated genes involved in cell division fidelity are unable to perform their duties. Thus, vinblastine treatment of irradiated cells induced the higher frequency of chromosome loss compared to the normal cells, which were represented as a higher frequency of MnBi.

In conclusion, the data of this study suggest that 2 Gy gamma irradiation not only has the main effect on genetic material seen as chromosomal abnormalities but also has subtle effects on genes involved in the division, which could sensitize the cells to other stimuli later in their life cycles. This finding has great importance in cases of radiation therapy and other procedures involved in cancer treatment. It has to be noted that cells of irradiated tissues might be more sensitive to other chemicals capable of inducing chromosome malsegregation. Such susceptibility would explain the high risk of second tumor formation in patient undergone radiotherapy.

## Conflict of Interest

The authors report no conflicts of interest.
